# The autobiographical IAT: a review

**DOI:** 10.3389/fpsyg.2013.00519

**Published:** 2013-08-13

**Authors:** Sara Agosta, Giuseppe Sartori

**Affiliations:** ^1^Center for Neuroscience and Cognitive Systems, Italian Institute of TechnologyRovereto, Italy; ^2^Department of Psychology, University of PaduaPadua, Italy

**Keywords:** implicit, associations, autobiographical memory, intentions, memory detection

## Abstract

The autobiographical Implicit Association Test (aIAT; Sartori et al., 2008) is a variant of the Implicit Association Test (IAT; Greenwald et al., 1998) that is used to establish whether an autobiographical memory is encoded in the respondent's mind/brain. More specifically, with the aIAT, it is possible to evaluate which one of two autobiographical events is true. The method consists of a computerized categorization task. The aIAT includes stimuli belonging to four categories, two of them are logical categories and are represented by sentences that are always true (e.g., I am in front of a computer) or always false (e.g., I am climbing a mountain) for the respondent; two other categories are represented by alternative versions of an autobiographical event (e.g., I went to Paris for Christmas, or I went to New York for Christmas), only one of which is true. The true autobiographical event is identified because, in a combined block, it gives rise to faster reaction times when it shares the same motor response with true sentences. Here, we reviewed all the validation experiments and found more than 90% accuracy in detecting the true memory. We show that agreement in identifying the true autobiographical memory of the same aIAT repeated twice is, on average, more than 90%, and we report a technique for estimating accuracy associated with a single classification based on the D-IAT value, which may be used in single subject's investigations. We show that the aIAT might be used to identify also true intentions and reasons and conclude with a series of guidelines for building an effective aIAT.

Autobiographical memory is the ability to remember events that constitute part of one's life, such as directly experienced events. It is part of the episodic memory, which is, in turn, part of the long-term memory (Tulving, 1983). Available assessment methodologies of autobiographical memories focus on the subject's overall ability to recall past memorized events. For example, the Autobiographical Memory Interview (AMI; Kopelman et al., 1989) consists of a series of questions asking subjects to retrieve personal events related to a target concept. Most techniques for investigating this field are limited to the estimation of the individual/patient's capacity of recalling past autobiographical information rather than measuring the presence/absence of a specific autobiographical memory.

Methods for evaluating single autobiographical memories are limited to a few techniques such as the Guilty Knowledge Test (GKT; Lykken, 1959; Ben-Shakhar and Elaad, 2003) also known as Concealed Information Test (CIT). The GKT largely relies on the orienting response. In a typical GKT examination, participants, while undergoing the polygraph testing (physiological measurements), are shown a series of stimuli, including a salient one, related to a crime. When the stimulus related to the crime is shown, the subject can easily recognize it, thus producing an orienting reflex (e.g., skin conductance increase and heart rate deceleration). For a recent book on this technique, see Verschuere et al. (2011).

A new method that can be used to identify a true autobiographical memory, intentions and reasons that motivate an act is the autobiographical Implicit Association Test (aIAT), a variant of the Implicit Association Test (IAT; Greenwald et al., 1998). Here, we will review all the published experiments on the aIAT so far. The traditional IAT (Greenwald et al., 1998) is a method for assessing the strengths of automatic associations. The method consists of a computerized task. Participants have to classify stimuli as quickly as possible in four different categories: two target concept categories (e.g., European American vs. African American names) and two attribute categories (pleasant vs. unpleasant) using two keys, one on the right and one on the left side of the keyboard. In one combined block, two categories (one from the target concept and one from the attribute dimension) are mapped on the same response key (e.g., European American names and pleasant words with the same key vs. African American names and unpleasant words with the other key). In a reversed combined block, participants have to classify the same four categories reversely paired (e.g., African American names and pleasant words with a key vs. European American names and unpleasant words with the other key), so that both target concept categories are paired with both attribute categories. The IAT effect is expressed as the difference between the combined and reversed combined blocks. In the block where two associated concepts require the same motor response, reaction times (RTs) will be faster than in the block where the same two concepts require different motor responses. Thus, the typical finding in this experiment is that, for European American participants, the stronger associated concept-attribute pair is the one coupling European American names and pleasant words: This block should be easier to categorize than the one associating African American names and pleasant words. The reversed pattern is found for African American participants. The IAT has been extensively studied in social psychology to assess implicit beliefs, attitudes, and prejudices to measure self-esteem and self-concept (Nosek et al., 2007).

Clinical applications indicate that the IAT may be an effective technique to identify suicide-prone subjects, Pedophilia sexual orientation, doping, and personality assessment (Gray et al., 2005; Schmukle et al., 2008; Nock et al., 2010; Petròczi et al., 2010). Nock et al. (2010), for example, reported that the IAT might be useful in detecting suicidal ideations in people who attempted suicide. The authors documented that a high implicit association between self and death in suicide attempters is linked to a 6-fold risk increase in committing a suicide attempt in the next 6 months.

The aIAT (Sartori et al., 2008) is a variant of the IAT (Greenwald et al., 1998) that could be used to establish whether an autobiographical memory trace is encoded in the respondent's mind/brain. More specifically, with the aIAT, it is possible to evaluate which one of two autobiographical events is true.

The aIAT differs, for example, from the above European American/African American IAT as the evaluative dimension (pleasant/unpleasant) is substituted by a logical dimension (True/False), which is represented by sentences describing events that are certainly true (e.g., I am sitting in front of a computer) and certainly false (e.g., I am climbing a mountain). Furthermore, the target concept categories (e.g., European American/African American) are represented by sentences describing alternative versions of an autobiographical event (e.g., I went to Paris for Christmas vs. I went to New York for Christmas), only one of which is true. The true autobiographical event is identified because, in a combined block, it gives rise to faster RTs when it shares the same motor response with true sentences. If the participant spent his/her vacation in Paris, the block associating true sentences and sentences related to Paris will be faster than the block associating true sentences and sentences related to New York.

The aIAT is structured in five blocks, three simple blocks (1, 2, 4), and two combined categorization blocks (3 and 5). In simple blocks, each response button is used to classify sentences related to only one category. In double blocks each response button is used to classify sentences related to two different categories.

In Block 1, participants have to classify true and false sentences (e.g., I am in front of a computer vs. I am in front of a television) using two response keys, one on the left and one on the right of the keyboard. In Block 2, participants have to classify autobiographical sentences (e.g., I went to Paris for Christmas vs. I went to New York for Christmas) with the same two response keys. In Block 3 (double categorization block), true sentences and sentences related to the first autobiographical event (e.g., Christmas in Paris) are paired on the same response key and false sentences and sentences related to the second autobiographical event (e.g., Christmas in New York) are classified with the other response key. In Block 4, only autobiographical events are reversely classified with the two response keys. Finally, in Block 5, participants have to classify both true sentences and sentences related to the second autobiographical event (Christmas in New York) with the same response key, and false sentences and the first autobiographical event (Christmas in Paris) with the other key.

The aIAT/IAT effect is expressed in terms of average RT difference between the two double categorization blocks: the congruent block (pairing the two associated categories) and the incongruent block (pairing the non-associated categories).

Used as a memory detection technique, the aIAT has a number of advantages related to the use of reaction times (Seymour et al., 2000), when compared to traditional psychophysiological techniques of lie detection (e.g., Ben-Shakhar and Elaad, 2003) or fMRI-based lie detection strategies (e.g., Langleben et al., 2005). For instance, it can be administered quickly (10–15 min), it is based on an unmanned analysis (no training for the user is necessary), it requires low-tech equipment (a standard PC is sufficient), and it can be administered remotely to many participants (e.g., via the internet).

## Detection of autobiographical memories: a review of validation studies

The aIAT accuracy in identifying the true memory has been investigated in a series of validation experiments summarized in Table 1. In this table, we separated first from second administration of an aIAT. Here, in order to evaluate the accuracy of the method, we included only experiments that did not include negative statements as subsequent investigations (Agosta et al., 2011c), conducted after the original publication (Sartori et al., 2008), indicated that the use of negative sentences or reminder labels generates unreliable and inaccurate results. For this reason, the following experiments were excluded:

1. Mock crime experiment (experiment 2) in Sartori et al. (2008).
2. Cocaine/heroine experiment (experiment 3) in Sartori et al. (2008).
3. Driving license experiment (experiment 5) in Sartori et al. (2008).
4. Control conditions (innocent or guilty) of experiments 1 to 3 reported by Verschuere et al. (2009).
5. Experiments 1 to 4 in Agosta et al. (2011c) used to verify the accuracy of the aIAT using negative statements.

**Table 1:** **In this table, the results from all the validation experiments are summarized**.

**Experiment**	**Source**	**Number of subjects**	**Participants classified correctly using the D-IAT**	**Average IAT effect**	**Average D-IAT**	**Confidence interval 95%**
**FIRST ADMINISTRATION**						
Card aIAT	Sartori et al., 2008	37	35/37	316 ms	0.56	0.45–0.67
			AUC = 0.99			
Holiday aIAT	Sartori et al., 2008	20	18/20	219 ms	0.44	0.25–0.63
Christmas holiday aIAT (non-faking group)	Agosta et al., 2011b	14	14/14	955 ms	1.06	0.84–1.28
Mock crime aIAT (affirmative sentences; first aIAT administered)	Agosta et al., 2011c	40	35/40	297 ms	0.56	0.41–0.71
Intention aIAT (Experiment 1)	Agosta et al., 2011a	22	22/22	712 ms	1.16	1.00–1.33
True memory aIAT (first aIAT administered)	Marini et al., 2012	18	18/18	879 ms	1.02	0.89–1.15
Flashbulb aIAT	Lanciano et al., 2012	42	Experiment 2 42/42	Experiment 2 876 ms	Experiment 2 1.48	Experiment 2 1.86–1.10
Experiment 1 = outlier		14	Experiment 1 14/14	Experiment 1 1082 ms	Experiment 1 3.87	Experiment 1 2.8–4.93
White lies aIAT (first aIAT administered)	Agosta et al., 2013	20	20/20	444 s	0.55	0.42–0.68
Reasons aIAT^*^ (first aIAT administered)	Agosta et al., 2013	20	20/20	309 ms	0.46	0.37–0.55
Mock crime aIAT	Hu and Rosenfeld, 2012	12 + 12 + 12 = 36	Immediate guilty: 10/12	Immediate guilty: 92 ms	Immediate guilty: 0.23	Immediate guilty: −0.08–0.54
			Innocents: 9/12	Innocents: 69 ms	Innocents: 0.32	Innocents: 0.13–0.51
			Delayed guilty: 7/12	Delayed guilty: 82 ms	Delayed guilty: 0.32	Delayed guilty: 0.14–0.50
Mock crime Pretest repetition group	Hu et al., 2012	16	16/16	121 ms	0.52	0.39–0.65
			AUC = 0.98			
Mock crime Pretest practice group	Hu et al., 2012	16	13/16	114 ms	0.46	0.29–0.63
			AUC = 0.91			
Mock crime Pretest instruction group	Hu et al., 2012	16	13/16	103 ms	0.47	0.28–0.66
			AUC = 0.95			
Mock crime Pretest training group	Hu et al., 2012	16	15/16	94 ms	0.51	0.35–0.67
			AUC = 0.98			
Action aIAT (Imagined + not imagined)	Takarangi et al., 2013	79	77/79	Not reported	0.585	0.53–0.64
Average	17 aIATs	412	92% accuracy		0.58	0.41–0.73
**SECOND ADMINISTRATION**						
Christmas holiday aIAT (non-faking group with previous aIAT experience not reported in the paper as was only considered a practice)	Agosta et al., 2011b	20	19/20	445 ms	0.64	0.48–0.80
Two cards aIAT (non faking group with previous aIAT experience not reported in the paper as was only considered a practice)	Agosta et al., 2011b	12	11/12	236 ms	0.45	0.26–0.64
Ten cards aIAT (non-faking group with previous aIAT experience not reported in the paper as was only considered a practice)	Agosta et al., 2011b	20	20/20	684 ms	1.13	1.03–1.22
Mock crime aIAT (affirmative sentences; second aIAT administered)	Agosta et al., 2011c	40	35/40	220 ms	0.45	0.33–0.57
True memory aIAT	Marini et al., 2012	18	18/18	606 ms	0.87	0.80–1.08
White lies aIAT (second aIAT administered)	Agosta et al., 2013	20	20/20	280 ms	0.45	0.34–0.56
Reasons aIAT (second aIAT administered)	Agosta et al., 2013	20	19/20	266 ms	0.50	0.39–0.61
Mock crime (Repetition group; second aIAT administered)	Hu et al., 2012	16	15/16	92 ms	0.41	0.28–0.54
Average	8 aIATs	166	94% accuracy		0.67	0.48–0.87

For each experiment, the number of participants together with average D-IAT values are reported. First administrations have been separated from second administrations of an aIAT.

* White lies and Reasons aIATs have been administered to the same participants, but have been included in this analysis not fulfilling the criteria for a systematic review. When excluding the Reason aIAT (second IAT administered to the same subjects), weighted average D-IAT is 0.59 for the first administration and 0.70 for the second administration. As shown, when eliminating the same subjects from analysis there are no substantial changes in the effect size.For each experiment, the number of participants together with average D-IAT values are reported. First administrations have been separated from second administrations of an aIAT.

Moreover, data used to calculate the accuracy refer to administrations of the aIAT prior to or without manipulations (faking, training, EEG-required-modifications of stimulus presentation) and for this reason we decided to exclude:

1. Naive faking and experienced faking groups in experiments 1–4 described in Agosta et al. (2011b).
2. Faking conditions of experiments 1–3 in Verschuere et al. (2009).
3. Intention aIAT combined with EEG (experiment 3 in Agosta et al., 2011a).
4. Second administration of the practice, instruction and training groups in Hu et al. (2012).

Repetitions of aIAT administrations to participants were only included in the analysis if there were no manipulations in between. Thus, in Table 1, we only report data from participants who either completed only one aIAT or two aIATs without manipulations in between.

In all the experiments, the validity of the aIAT was tested against a known false event. For example, in the card experiment, a card, which was actually chosen by the participant, was compared to the non-selected card. In the autobiographical memory experiment, a real autobiographical event, as assessed through a preliminary questionnaire, was compared to a false event. For this reason, we excluded:

1. Experiment 2 in Agosta et al. (2011a) because evaluating the difference between intentions and hopes and not presenting two contrasting events or intentions (i.e., hopes are true as well as intentions).
2. False memory aIAT in Marini et al. (2012) because comparing two actual false events (one believed to be true).

Two measures can be used for evaluating the diagnostic accuracy: the magnitude of the IAT effect (RTs of the incongruent block minus the RTs of the congruent block) and the D-IAT value (D600; Greenwald et al., 2003). Here, we focused in particular on the D-IAT value. This index combines speed of response and classification accuracy. It includes a penalty for errors and variability. It expresses the difference in the mean latencies of the double categorization blocks scaled by the standard deviation of response latencies. It is calculated by subtracting corrected mean RTs of the congruent block from corrected mean RTs of the incongruent block and dividing this difference by the inclusive standard deviation for the two blocks.

Effect size was the average D-IAT value. To calculate an average effect size across all the studies, the D-IAT values were weighted by the inverse variance in order to deal with the different and small sample sizes of each study (Lipsey and Wilson, 2001). The only outlier (Flashbulb aIAT, Experiment 1; Lanciano et al., 2012) was identified using the interquartile range and was not included in the calculation of the mean effect size.

For the first-administration studies (17), homogeneity among study results was evaluated using Cochran's Q combined with the *I*^2^ statistic. Cochran's Q value had to be compared to a chi-square distribution with k-1 (number of studies -1) degrees of freedom. In our case, it resulted in a value of 9.26, below the critical value for 16 degrees of freedom in a chi-square distribution (26.3). This value indicated low heterogeneity. The interpretation of the *I*^2^ statistic was made following Higgins and colleagues' directions (Higgins et al., 2003) with values of 25% representing low heterogeneity, 50% moderate heterogeneity, and 75% high heterogeneity. Our *I*^2^ is equal to 0%. D-IAT values were combined to obtain a mean effect-size using a fixed-effect approach because of the low heterogeneity. D-IAT average value resulted in 0.57 (95% C.I. 0.41–0.73).

For a total of 8 s administration studies, Cochran's Q was 8.51 (<14.1–7 degrees of freedom) and the *I*^2^ was 0%. Again, we used a fixed-effect model for calculating the mean effect size of 0.67 (95% C.I. 0.48–0.87).

Weighted average D-IAT for the first administration was 0.57, while for the second-administration studies it was 0.67. More studies are needed in order to investigate the effect of repetition of an aIAT. Indeed, in the studies reported here, the same aIAT has never been repeated twice.

To determine the accuracy of the test, we used the direction of the D-IAT values, calculated by subtracting the congruent block from the incongruent one, with negative values indicating an incorrect classification (i.e., the identification of the false memory as true) and positive D-IAT values indicating the correct identification of the true memory.

Accuracy was also calculated across a total of 412 first administrations of the aIAT to participants (*Q* = 4.7 < 26.3; *I*^2^ = 0%). The weighted average classification accuracy was 92% (95% C.I. 83–100%). Across a total of 166 s administrations (*Q* = 0.41 < 14.1; *I*^2^ = 0%), the weighted average accuracy was 94% (95% C.I. 80–100%). Clearly, repetition of the aIAT does not decrease the overall accuracy.

In this small review, we mainly included experiments from the same laboratory. Importantly, in Table 1, we have also included data of five mock-crime experiments from two other laboratories (Hu and Rosenfeld, 2012; Hu et al., 2012; Takarangi et al., 2013). These data include preliminary aIATs administered to four groups of participants that were subsequently tested with a variety of manipulations between test and retest, and data on performed and non-performed actions. Finally, we added data from an associated laboratory (Lanciano et al., 2012).

Recently, a modified version of the IAT/aIAT has been used in order to distinguish between seen and unseen events (eyewitness—Implicit Association Test—eIAT; Freng and Kehn, 2012). The authors tested a total of 18 participants and showed that the eIAT “successfully distinguished between witnessed and non-witnessed details” of a video. In particular, they reported that central and peripheral details of a scene were efficiently identified (central details; *D* = 0.5, peripheral details *D* = 0.42). These data have not been included in Table 1 because of a lack of details in the text (i.e., average reaction times of congruent and incongruent blocks, accuracy of the D-IAT values in identifying the eye-witnessed event). Results of this experiment show that the aIAT cannot only be used to identify episodic memory of an own action, but also an observed event.

## Overall accuracy and accuracy as a function of D-IAT

The D-IAT value measures the strength of the IAT effect combining both RTs and errors. The D-IAT value used as classification criterion yields correct classifications in more than 90% of the cases, with a weighted average value of 0.58 for first-administration studies and 0.67 for second-administration studies.

**Figure 1:**
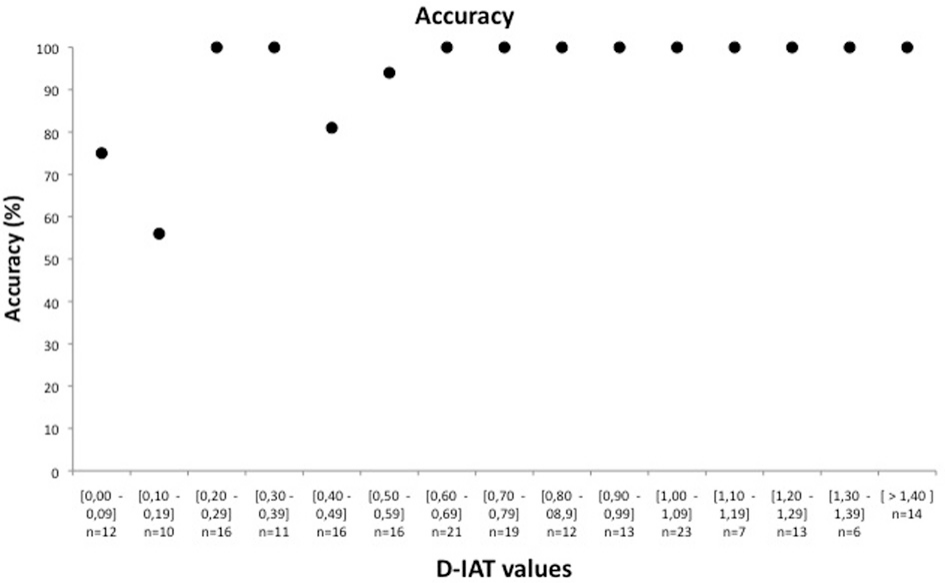
**Classification accuracy as a function of the D-IAT value**. Data from eight validation experiments, for a total of 209 subjects, were used to calculate accuracy in identifying the true autobiographical memory on the basis of the D-IAT value. D-IAT values have been grouped in bins of 0,1. In the Y axis, the number of participants for each bin is reported.

When analysing the relation between classification accuracy and D-IAT values, we found that it varies depending on D-IAT values. For D-IAT values just above zero, classification accuracy is just above 50%, while for D-IAT values larger than 0.6, the classification is almost 100% (please refer to Figure 1).

Figure 1 was drawn as follows:

1. Data were used from eight previous validation experiments [first-administration aIAT only and limited to experiments conducted in our research group: Card aIAT in Sartori et al. (2008); Holiday aIAT in Sartori et al. (2008); Christmas holiday aIAT in Agosta et al. (2011b); Mock crime aIAT in Agosta et al. (2011c); Intention aIAT in Agosta et al. (2011a); True and false memory aIAT in Marini et al. (2012); White lies aIAT in Agosta et al. (2013); Reasons aIAT in Agosta et al. (2013)] for a total of 209 subjects.
2. For each participant, the D-IAT value was used with information about whether the classification of the target true autobiographical memory was correct (1) or wrong (0).
3. Subjects were ordered for increasing values of absolute D-IAT value.
4. D-IAT values were grouped in bins of 0.1.
5. For each D-IAT value group, the corresponding accuracy was calculated (different number of participants for each D-IAT value group).
6. Each black dot on the figure represents the corresponding accuracy of a specific D-IAT value group.

This D/accuracy figure highlights the close relationship between accuracy and D-IAT value. First, it is important to note that only for a few D-IAT values is the accuracy lower than 0.8 (80% correct classifications), and most of the values with the lower accuracy are included in the window between 0 and 0.2. For this reason, we would advise considering any D-IAT value from 0 to 0.2 as inconclusive. Across the total of 209 subjects, 10% showed an inconclusive result. Moreover, the figure highlights the fact that D-IAT values greater than 0.6 are always classified correctly and, more importantly that the majority of the D-IAT values have 100% accuracy. This D/accuracy function could help in estimating the probability of correct classification depending on the individual test result, thus increasing the confidence of the technique when making inferences on a single test.

An important issue in clinical and forensic single-case investigations is the estimation of the validity of the test results. In short, if a subject's D is equal to 0.43 from Figure 1, we would expect that his/her result is in the 0.4–0.49 range and has an average accuracy of 81%.

## Reliability of aIAT

### Split-half reliability

Ideally, a good memory detection technique should identify the same true memory from different subsets of items. This feature is assessed with the split-half technique.

aIAT split-half reliability has been computed after separating odd and even stimuli and then deriving, for each test, two D-IAT values. Data were calculated over a subgroup of the previous validation experiments: the first-administration studies. The main result indicates an average 88% of agreement in the identification of the true autobiographical memory (correct or incorrect classification of the subject on the basis of the D-IAT value), of even and odd stimuli (please refer to Table 2).

**Table 2:** **Split-half correlation, percentage agreement between classifications derived on even numbers and classification derived from odd numbers in five experiments**.

**Experiment**	**Agreement %**	**Split-half correlation**
Card aIAT (Sartori et al., 2008)	73	*r* = 0.47 *p* < 0.003
Mock Crime (Agosta et al., 2011c)	85	*r* = 0.48 *p* < 0.002
Christmas holiday aIAT (non-faking group, Agosta et al., 2011b)	100	*r* = 0.67 *p* < 0.006
Intentions (Experiment 1, Agosta et al., 2011a)	90	*r* = 0.17 *p* = 0.465
White lie aIAT (Agosta et al., 2013)	90	*r* = 0.83 *p* < 0.001

Correlations of the D-IAT values, calculated separately for odd and for even trials, resulted in an average split-half value of *r* = 0.52, with a low correlation between even and odd stimuli in the “Intention aIAT.” There are no apparent reasons for this low correlation, but the agreement in identifying the true autobiographical memory is 90%. Thus, even if the correlation of the D-IAT values is low, both values, derived from the even and odd stimuli, result in a comparable identification of the true autobiographical memory.

### Order of presentation of the congruent block

In order to establish if there is an agreement between the results obtained with different orders of the congruent and incongruent blocks (3rd and 5th positions), we also analysed the correlation of D-IAT values of the same aIAT with the congruent block either in the 3rd (direct order) or the 5th position (reversed order), and consequently, the incongruent block in the 5th or the 3rd position. Two experiments (Table 3) in which participants were administered both orders (direct and reversed), taken from the previous validation table, were used for this analysis: the “Mock crime” aIAT reported in Agosta et al. (2011c) and the “White lie” aIAT (Agosta et al., 2013). All the participants in the two experiments were administered two aIATs: one in the direct and one in the reversed order. In the “Mock crime” aIATs, the order of presentation of the two aIATs was counterbalanced across subjects, while in the “White lies” aIAT (Agosta et al., 2013) the first aIAT always had the congruent block in the third position.

**Table 3:** **Correlation and agreement of D-IAT values and IAT effect for normal (congruent block in 3rd position and incongruent block in 5th position) and inverted (congruent block in 5th position and incongruent block in the 3rd position) orders**.

**Experiment**	**3–5**	**Agreement D-IAT**	**Correlation IAT effect**	**Correlation D-IAT**
Mock crime (Agosta et al., 2011b)	Order counterbalanced across participants	85%	*r* = 0.25 *p* < 0.11	*r* = 0.63 *p* < 0.001
White lies (Agosta et al., 2013)	Order fixed with the first aIAT administered having the congruent as block 3 and the second aIAT administered having the congruent block as block 5	95%	*r* = 0.25 *p* < 0.28	*r* = 0.15 *p* < 0.53

Results indicated that the agreement in the identification of the true autobiographical memory for the direct and reversed orders (on the basis of the direction of the *D* values) was high: 95% and 85% for the “White lie” and “Mock crime” experiments, respectively.

Moreover, the correlation of D-IAT values (for the direct and reversed orders) was 0.15 for the “White lie” and 0.63 for the “Mock crime” experiment. For the White lie experiment, as for the Intention experiment presented in the previous section, we do not have an explanation for this low correlation, but the level of agreement is high and there is no reduction in the identification of the true memory.

## Factors reducing accuracy and modulating the aIAT

Further research was conducted in order to highlight the limitations in the use of aIAT. Specifically, the effect of faking, of using negative sentences and negative labels, has been investigated. The results are summarized below.

### Effects of faking on memory detection

Verschuere et al. (2009) have shown that properly trained participants may alter the test outcome strategically. Participants may be trained to alter the test outcome by speeding up the incongruent blocks and slowing down the congruent block. Verschuere et al. (2009) instructed the guilty participants in a mock-crime task to appear as “innocents” by slowing down their responses. Their results indicated that a big percentage of the guilty participants not previously exposed to the aIAT succeeded in faking the test, but only when explicitly taught the strategy to counterfeit the test outcome. These results were further refined by Agosta et al. (2011b) who showed that: (i) instructed fakers (explicitly instructed by the experimenter to succeed in altering the test outcome) may alter the test outcome by making a false memory appear true and vice versa and (ii) fakers may be distinguished from non-fakers on the basis of an algorithm that compares response speed in simple blocks with response speed in double blocks. Their results are summarized in the Table 4.

**Table 4:** **Data from four experiments comparing control non-fakers, naïve fakers, and instructed fakers are reported (Agosta et al., 2011b)**.

**Experiment**	**Non-fakers D-IAT**	**Non-fakers correct classifications**	**Naïve fakers D-IAT**	**Naïve fakers correct classifications**	**Instructed fakers D-IAT**	**Instructed fakers correct classifications**
1	1.06	14/14	0.78	14/14	−0.45	5/14
2	0.64	19/20	0.24	6/10	−0.42	7/20
3	1.13	20/20	0.82	18/18	−0.81	4/34
4	0.45	11/12	0.15	6/12	0.06	7/12

Control non-fakers were administered the test without specific instructions, and naïve fakers were instructed to alter the results but were not taught the more efficient strategy. Instructed fakers were instructed to alter the results by speeding up in the incongruent trial and slowing down in the incongruent trial.

In short, a non-trained subject instructed to fake, but using self-discovered strategies, does not often succeed in his/her attempt. By contrast, when previously trained on the best strategy to fake (e.g., speed up incongruent block and slow down the congruent block), examinees can alter their results and beat the “memory detector.” However, these successful fakers may be detected on the basis of their response pattern through a faking-detection algorithm. This algorithm is based on a comparison of the average speed in double and single blocks. Indeed, participants leave a signature when trying to fake the test: They do not alter their RTs in single blocks and are abnormally slow in double categorizations blocks (Agosta et al., 2011b). This feature has been used with high accuracy (83%) to detect fakers. The more efficient algorithm for detecting fakers consists of three steps: (i) remove all responses below 150 and above 10,000 ms, (ii) replace errors with the average RT of the block with a penalty of 600 ms, and (iii) calculate the ratio between the average RT of the fastest block (between 3 or 5) and single tasks that are directly connected to the fastest task in terms of motor response (1 and 2 or 1 and 4, respectively). If the result exceeds 1.08, then the respondent is faking. This cut-off was identified as the one yielding the maximal classification accuracy in our sample.

Hu et al. (2012) investigated this same issue. The authors confirmed that specific instructions given to the subjects might be effective in altering the aIAT results. Furthermore, they showed that this pattern of results might be further enhanced with a specific training in the incongruent trial. Thus, they reported that instructions and training together are more effective than instructions alone in reversing the results compared with a pre-test. In their experiment, they reported failing to find a significant difference between fakers and non-fakers using the previously described indexes. Those results highlight the need for an in-depth investigation of this important issue. Only two studies have been published so far on the possibility of identifying fakers with non-consistent results.

### Effects of negative sentences as descriptors of autobiographical events on aIAT accuracy

False memories may be described by using a negative description of the true memory. Agosta et al. (2011c) have shown that, when affirmative sentences and reminder labels are used to describe the true and false autobiographical events, accuracy is very high at up to 90% (Agosta et al., 2011c). By contrast, in four studies, the authors (Agosta et al., 2011c) showed that when negative sentences and labels are used, there is a reduction of about the 30%, in the accuracy of the aIAT in identifying the true autobiographical event. The accuracy of the aIAT is reduced not only by negative sentences, but also by affirmative sentences describing counter-events. The affirmative counter-event sentences were stated with expressions such as *different place from* instead of the negative (e.g., “I have been to Rome,” vs. “I have been to a different place than Rome”). Negative and affirmative counter-event sentences can be considered as equivalent from this point of view. Counter-event sentences show a more difficult grammatical structure than simple negative sentences and, at the same time, have a negative inner meaning (e.g., having been in a different place than Rome means not having been in Rome). Those might be plausible reasons for the aIAT's low accuracy when using counter-event sentences. The use of negatives renders the test highly inaccurate and should therefore be avoided.

## aIAT application to flashbulb and false memories

### Flashbulb memories

For many years, researchers have debated whether flashbulb memories (FBMs) can be considered either as a special class of accurate emotional memories that are exceptionally vivid and resistant to decay (Pillemer, 1984; Bohannon, 1988; Conway et al., 1994) or as memories affected by reconstructive factors such as ordinary autobiographical memories. The controversial debate concerning the real existence of this special class of memories reflects the difficulty in establishing the accuracy of these autobiographical formations.

FBMs are usually recalled with a higher degree of confidence than other autobiographical memories (Brown and Kulik, 1977; Weaver, 1993; Talarico and Rubin, 2003, 2007, 2009). It is interesting to note that the participants' confidence does not decrease even when it is clear that the recalled event had not occurred in the same way as it is remembered (Neisser and Harsch, 1992). Indeed, according to some authors, what makes FBMs so unique and special is the individual's sense of confidence in his/her accuracy, which is preserved for a long time after the occurrence of the original eliciting event (Weaver, 1993; Talarico and Rubin, 2003, 2007, 2009).

Lanciano et al. (2012) have investigated the specific characteristic of FBM by asking 14 participants to fill out a questionnaire concerning the death of Pope Johannes Paulus II. On average, subjects were tested 2235 days after the pope's death. The questionnaire investigated seven FBM attributes: (1) date when the individuals learned of the pope's death, (2) day, (3) time of the day, (4) informant (family, friends, colleagues, media), (5) location (country, city, room, or other kind of location, i.e., the car), (6) presence of other people, and (7) ongoing activity. An aIAT contrasting the true memory with a fabricated false memory was administered to participants 1 week later. All 14 participants were correctly classified using the D-IAT values. Average D was 3.85, which is a very high value compared to other typical values as reported in Table 1. Consistency among repeated measures of FBM is a typical parameter describing the quality of this sort of memory. The authors reported a high correlation of 0.85 between consistency value and D-IAT values at the aIAT. In short, the more consistent the FBM is among repetitions, the higher the D-IAT value is observed.

### False memories

It is known that human memory is prone to various kinds of distortions and illusions (Roediger, 1996; Schacter, 1999; Loftus, 2003). It has been shown that, in contrast to deception, memory illusions are often not accompanied by a subjective feeling that people are responding untruthfully. Quite the contrary, memory illusions like those produced in the Deese-Roediger-McDermott paradigm (DRM; Deese, 1959; Roediger and McDermott, 1995) are accompanied by a sense of recollection that, at the conscious phenomenological level, makes them indistinguishable from true memories. DRM false memories are obtained by presenting lists of words related to a non-presented critical lure. The probability of recalling and recognizing the critical lure is usually quite high (Roediger and McDermott, 1995; Balota et al., 1999; Stadler et al., 1999; Budson et al., 2002). Previous findings have shown that critical lures seem to elicit the same quality (i.e., remember judgments) of presented items (e.g., Roediger and McDermott, 1995), and participants are even able to state in which voice they heard the non-presented critical lure when half of the list items had been presented by a female voice and half by a male voice (Payne et al., 1996).

In this study, Marini et al. (2012) used a standard DRM task to induce false memories, followed by the two aIATs. By comparing the results of the two aIATs, one could observe whether participants were responding differently to true and false DRM memories. One aIAT compared presented items with non-presented distracters (aIAT true memories), whereas the second aIAT (aIAT false memories) compared critical lures with non-presented distracters. Specifically, the aIAT true memories evaluated the association of the presented items with the true logical dimension, while the aIAT false memories evaluated the association of the critical lures with the true logical dimension. Therefore, if true memories (presented items in the aIAT true memories) and false memories (critical lures in the aIAT false memories) were encoded differently, as suggested by neuroimaging studies (Cabeza et al., 2001; Slotnick and Schacter, 2004), they would have a different strength in their association with the true logical dimension. If, however, the aIAT is based on the individual's “aware” belief that the critical lure is indeed present, then the aIAT would be ineffective in detecting any difference between presented items and critical lures. Results indicated that false memories are strongly associated with true sentences (36/36 participants), giving rise to similar associations as true memories with true sentences.

This result indicates that the aIAT reflects exactly what is stored in our memory, and if a memory is strongly believed to be true, then the aIAT would identify it as a true memory. An interesting issue that stems from the false-memory work concerns its applied implications: Does the aIAT always identify a true memory when this is strongly believed to be true? Does the self-persuasion of a false memory as true influence the result of the aIAT? All these issues have to be investigated in more detail in future studies.

It has been shown that false memories may stem from “source confusion” (Takarangi et al., 2013), which is defined as “the attribution of a specific memory to a particular source using heuristics that may lead to errors.” Takarangi et al. (2013) reported an experiment aimed at verifying the aIAT diagnostic abilities in detecting whether an action was performed or not. After asking their participants to perform or not to perform an action, the authors further required them to imagine both performed and non-performed actions.

They reported an overall aIAT accuracy of 97.5% in detecting whether the action was performed or not, confirming the efficiency of aIAT in identifying memories of performed actions when contrasted to memories of non-performed actions.

Importantly, the experimental design allowed the computation of a source discrimination score derived by subtracting ratings for non-performed actions from ratings of performed actions (the authors asked the participants to rate how much they believed that they had performed the action, and then rated how much they remembered performing the action). They found that imagining an action increased the subjective trend of believing and remembering actions as performed rather than non-performed actions. They also found that the D-IAT value diminishes with the source discrimination score. In short, the more the memories are subjectively confused (acted vs. not acted) by the subject, the lower the D-IAT. The authors claim that this is a limitation of the aIAT when it is required to identify false memories. However, a close inspection of Figure 2 in their paper shows that only two of 79 subjects were misclassified and this indicates that, even if D-IAT is affected by source confusion, this did not increase misclassification in their study.

## Detection of intentions

Deliberation of a future action is called prior intention in one terminology (Searle, 1983). Prior intentions include goal-related processing and deliberative conscious intentions that are intuitively believed to be the leading cause of our future behaviors (Bratman, 1987; Cohen and Levesque, 1990). In other words, these are mental representations that occur prior to the action itself and are typically believed to cause the action subjectively. Searle (1983) refers to prior intentions as the initial representation of the goal of an action prior to the initiation of the action: a type of intention that is formed in advance of a deliberate plan for a future action. In contrast, an intention in action (also termed motor intention) is the proximal cause of the physiological chain leading to an overt behavior.

Other scholars have addressed a possible distinction between long-term antecedents of action (prior intentions; Searle, 1983) and short-term antecedents of actions (intentions in action; Searle, 1983; Becchio et al., 2008, 2010). Long-term antecedents have also been named “prospective intentions” (Pacherie and Haggard, 2010), “distal intentions” (Pacherie, 2008), or “future-directed” intentions (Bratman, 1987).

An experiment showing that intentions to act may be identified reliably with the aIAT will be summarized here (please refer to Table 5). Agosta et al. (2011a) have investigated whether real intentions could be distinguished from false intentions using the aIAT, finding that both short-term intentions (where to sleep the upcoming night) and long-term intentions (professional career) could be distinguished from plausible, but false intentions.

**Table 5:** **The data for the intention experiment**.

**Experiment**	**Participants**	**RT congruent (ms)**	**RT incongruent (ms)**	**D**	**% correct classification**
Short term—sleep	*N* = 11	1011	1975	1.30	11/11
Long term—job	*N* = 11	1047	1507	1.02	11/11

The first condition refers to a short-term intention, where to sleep the coming night, while the second condition refers to a long-term intention of future work. Classification reaches 100% accuracy for both conditions.

They further showed that the basis of such discrimination was related to intentions *per se* rather than hopes. In fact, when contrasted with hope sentences, intentions with or without pleasant outcomes were strongly associated with true sentences (Agosta et al., 2011a; Experiment 2).

## Detection of reasons underlying lies

According to De Paulo et al. (1996) and Vrij (2007), the reasons to lie may differ in terms of (1) the person who benefits from the lie (whether self or other-oriented), (2) the consequences of lying (in order to gain advantage or to avoid costs), and (3) the type of lying (whether for materialistic or psychological reasons). Self and other oriented lies are told either to protect oneself or others psychologically (e.g., protect from embarrassment or loss of face). According to Feldman (2009), standards of tact and politeness and expectations can make deception, to some degree, almost inevitable. Agosta et al. (2013) showed that the aIAT might be used to distinguish true from false reasons underlying other oriented lies (white lies) and that 20/20 (direct order) and 19/20 (reversed order) participants were correctly classified, with a D-IAT average value of 0.46 (direct order) and 0.50 (reversed order), respectively.

## Conclusions

We have reviewed the validation experiments conducted so far that use the autobiographical IAT. The aIAT is a variant of the IAT (Greenwald et al., 1998) that might be used to establish the association of an event with the true/false logical dimension. In other words, the aIAT reveals which one of two contrasting events is more associated with the truth.

Validation experiments have highlighted high classification accuracy over a series of tests with average accuracy over 90%. The average effect sizes were moderate: 0.57 for first-administration experiments and 0.67 for second-administration experiments. The previous results refer to a wide range of type of memories for a total of 578 subjects. Results from Experiment 1 in Lanciano et al. (2012) were excluded because the D-IAT was abnormally high, presumably due to the outstanding features of flashbulb memories.

It is worth noting that the same research group has carried out most of the studies conducted so far. Only a few experiments were conducted outside our laboratory (e.g., Hu and Rosenfeld, 2012; Hu et al., 2012; Takarangi et al., 2013) or in one associated laboratory (Lanciano et al., 2012). More studies from other laboratories are needed in order to better validate the technique and to determine a more reliable effect size, as some of the independent replications revealed lower effect sizes (Hu and Rosenfeld, 2012; Hu et al., 2012).

The validity of other lie detection techniques such as the CIT has usually been calculated using Cohen's d. For example, the meta-analysis by Ben-Shakhar and Elaad (2003) reported an overall average effect size of *d* = 1.55. Comparison of aIAT and CIT effect sizes test might be difficult, given substantial differences in calculating Cohen's *d* (calculated as the difference between the means of the detection score distributions of the guilty and innocent samples; Ben-Shakhar and Elaad, 2003) and the D-IAT values (calculated as the difference between the incongruent and congruent blocks of the same aIAT). The D-IAT algorithm takes into account the phenomenon of speed-accuracy trade-off, which is not an issue in CIT experiments.

In the future, the validation pipeline should include test-retest reliability over longer time frames and all other issues addressed in the CIT/GKT literature such as the modulating effects of personality and the full investigation of countermeasures. The CIT is the major memory-detection technique and has a much longer history and has been tested on a wider variety of conditions. The aIAT validation studies, compared with the CIT validation studies, lack extensive field studies. As is frequently reported, in the lie detection literature, studies carried out in the laboratory tend to overestimate accuracy and for this reason it will be critical for the aIAT to collect data in more ecological high-stake conditions (Elaad, 2011).

We have also identified a series of conditions that reduce the validity of the test and therefore should be avoided. Such conditions include the use of negative sentences in describing the events as well as using negative reminder labels. We have derived a D/accuracy function that permits us to estimate at the single subject level the probability of a given result in terms of accuracy, showing that classification accuracy for D-IAT values in the range of 0–0.2 is very poor, while D-IAT values above 0.6 are high and values between 0.2 and 0.6 are above 80%. In practical uses of the aIAT, attention should be paid to the level of D-IAT size as an indirect index of result reliability.

Here we summaries the guidelines for building an effective aIAT on the basis of the validation experiments reported above:

- Sentences related to true and false categories should always be true and false for the respondent (examples of *true* sentences are “I am in front of a computer,” or “I am sitting on a chair”; examples of *false* sentences are “I am climbing a mountain,” or “I am skiing”).
- Only one of the two events used to build an aIAT should be true and the other should be false. Two contrasting events should always be used; for example, “I left the door open” and “I closed the door” are good examples of sentences, as only one of the two is true for the respondent. The aIAT is supposed to uncover which one of the two is true.
- Do not use negative reminder labels or sentences. Use two contrasting autobiographical events.
- Before proceeding to the interpretation of the results, check whether the examinee has faked the test or not. The only available index up to now has been published in Agosta et al. (2011b). It compares response speed in single blocks (blocks 1, 2, 4) with response speed in double blocks (blocks 3 and 5).
- Evaluate which of the two autobiographical sentences is associated with *true* sentences using the D-IAT value. Compare blocks 3 and block 5. Identify the fastest block. The target autobiographical memory as the one that is more associated with the *true* logical dimension (on the basis of the faster reaction times in blocks 3 or 5).
- In single case studies, the use of a window of uncertainty is recommended. We suggest 0–0.2 as the uncertainty window. In such a range, probabilities of correct classification range from 50 to 75%. Reliability, as measured by agreement, is good for D-IAT values between 0.2 and 0.6 and very good for D-IAT values above 0.6.

Here, we add suggestions for aIAT users resulting from our own experience and highlighting the need for new studies that deeply investigate these issues:

- Memories should be encoded in sentences limited to a single line and about half a screen.
- Sentences describing autobiographical events should give a clear-cut description of the event. Fuzzy descriptions should be avoided, as valid discriminations have not been proven for such types of descriptions.
- Only two single, specific events (one true and one false) should be investigated; no more than two events, even if grouped in the same categories, should be used.
- In single case studies, confidence in the final results could be enhanced by using a design that includes: (i) build an aIAT on known autobiographical data (e.g., date of birth, names of sons, etc.) and check that this personal information is correctly identified and (ii) when testing the event of interest, the central fact and peripheral details should be used in different aIATs. The administration of these different aIATs should be delayed in time at least one week from one administration to the other in order to avoid any reduced effect on the D-IAT value.

### Conflict of interest statement

The authors declare that the research was conducted in the absence of any commercial or financial relationships that could be construed as a potential conflict of interest.
